# Single‐Stage Modified Postauricular Flap Reconstruction for a Traumatic Helical Rim Defect Following a Human Bite: A Case Report

**DOI:** 10.1002/ccr3.72790

**Published:** 2026-06-05

**Authors:** Khaled Mohamed AlAani, Ahmed Alanzi, Madhusudana Naik Mude, Dawood Alatefi, Ahmed M. Kazerooni, Ali Mahran Kazerooni, Noor Mohamed AlAani

**Affiliations:** ^1^ College of Medicine and Medical Sciences Arabian Gulf University Manama Kingdom of Bahrain; ^2^ Anaesthesia & Pain Management Department King Hamad University Hospital Muharraq Kingdom of Bahrain; ^3^ Consultant Plastic Surgeon, Bahrain Specialist Hospital Manama Kingdom of Bahrain; ^4^ The University of Jordan Amman Jordan; ^5^ Division of Plastic and Reconstructive Surgery, Department of Surgery Mayo Clinic Rochester Minnesota USA; ^6^ Royal College of Surgeons in Ireland – Medical University of Bahrain Muharraq Kingdom of Bahrain

**Keywords:** auricular trauma, ear defect, helical rim reconstruction, human bite injury, postauricular flap

## Abstract

Traumatic defects of the helical rim caused by human bites present significant reconstructive challenges because of tissue loss, infection risk, and the need to preserve auricular contour and symmetry. We report a 27‐year‐old man who presented with a middle‐third defect of the left helical rim following necrosis of an initially sutured avulsed flap caused by a human bite injury. After discussion of reconstructive options, including chondrocutaneous advancement and staged flap procedures, a single‐stage modified postauricular flap without skin grafting was selected to preserve ear size and minimize donor‐site morbidity. The flap was successfully inset with restoration of the helical contour, and postoperative follow‐up demonstrated stable healing, preserved auricular shape, and high patient satisfaction without complications. This case highlights that a single‐stage modified postauricular flap is a reliable and patient‐centered option for reconstruction of traumatic helical rim defects following human bite injuries.

## Key Clinical Message

Human bite injuries of the helical rim may cause tissue necrosis and complex auricular defects. In selected patients, a single‐stage modified postauricular flap can restore helical contour, preserve ear size, avoid donor‐site skin grafting, and achieve satisfactory aesthetic outcomes.

## 1 Introduction

Human bite injuries to the external ear are relatively uncommon but can result in significant tissue loss and aesthetic deformity because of the ear's exposed anatomical position and the high bacterial load associated with human saliva. Such injuries carry a substantial risk of infection, tissue ischemia, and necrosis, frequently leading to failure of primary repair and the need for secondary reconstructive procedures [1, 2]. The helical rim represents a critical structural and aesthetic component of the auricle, and even small defects in this region can disrupt the natural curvature of the ear and compromise overall facial symmetry [3].

A variety of reconstructive techniques have been described for helical rim defects, ranging from primary closure and local chondrocutaneous advancement flaps to regional and staged flap procedures. The Antia–Buch chondrocutaneous advancement flap remains one of the most widely used methods for reconstruction of marginal helical rim defects because it allows advancement of well‐vascularized local tissue while preserving the natural contour of the auricle [4]. However, this technique may reduce auricular circumference and may not be suitable for larger defects or cases in which preservation of ear size is a priority [5].

Alternative reconstructive options include postauricular flaps, temporoparietal fascial flaps, and staged tubed flaps, which provide additional tissue coverage but may involve multiple procedures or increased donor‐site morbidity [5, 6, 7]. Contemporary reconstructive strategies increasingly emphasize individualized surgical planning that considers defect characteristics, tissue availability, and patient preferences in order to achieve optimal functional and aesthetic outcomes [7, 8].

In this report, we present a case of a traumatic middle‐third helical rim defect following a human bite injury that was successfully reconstructed using a single‐stage modified postauricular flap. This case highlights the role of patient‐centered reconstructive decision‐making and demonstrates how a modified local flap technique can restore auricular contour and symmetry while avoiding staged procedures and donor‐site skin grafting.

## 2 Case Presentation

### 2.1 History and Physical Examination

A 27‐year‐old man presented with a deformity of the left helical rim following a human bite injury sustained during a physical altercation. The initial avulsed segment had been sutured several hours after the injury, but this flap subsequently developed ischemia and necrosis, resulting in a well‐demarcated middle‐third defect of the left helical rim. The patient was subsequently referred to our surgical service several days after the initial trauma for evaluation and reconstruction. The patient's main concern was the visible deformity of the ear, which was both aesthetically displeasing and psychologically distressing. He had no significant past medical history, no family history of relevant disease, no drug allergies, and no social habits that could adversely affect wound healing.

**FIGURE 1:**
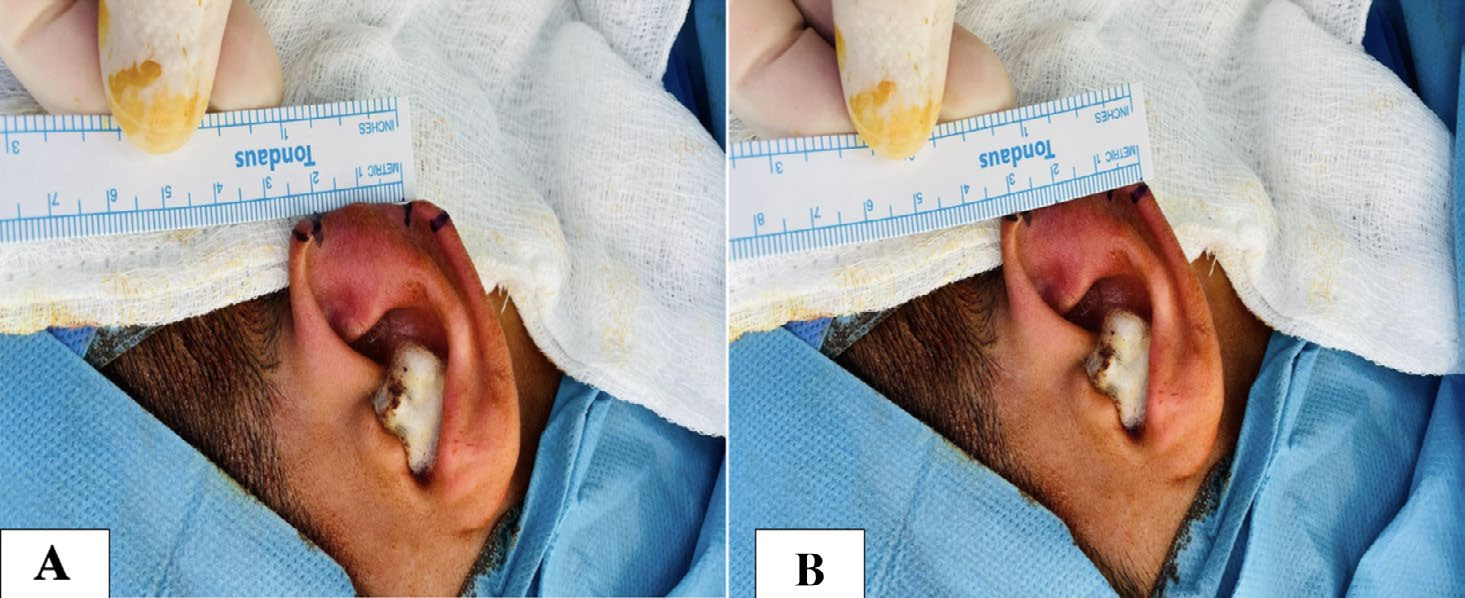
Preoperative appearance of the left ear showing a middle‐third helical rim defect following a human bite injury. (A) Measurement of the defect along the inner margin of the auricle (2.5 cm). (B) Measurement of the defect along the outer margin of the auricle (3.5 cm).

On physical examination, there was a clearly defined defect involving the middle third of the left helical rim with intact surrounding tissue and no signs of active infection or inflammation. Preoperative assessment demonstrated that the defect measured approximately 2.5 cm along the inner margin and 3.5 cm along the outer margin of the auricle (Figure 1A,B).

### 2.2 Investigations and Treatment

Laboratory investigations were within normal limits, and no radiological imaging was performed because the injury was confined to the superficial soft tissues. The clinical diagnosis was traumatic helical rim loss due to flap necrosis following a human bite injury, based on the patient's history, physical examination, and progressive ischemia of the initially sutured tissue.

Because the injury resulted from a human bite, appropriate infection prevention measures were implemented. The wound was thoroughly irrigated and debrided during the initial management, and the patient received prophylactic antibiotic therapy with amoxicillin‐clavulanate to cover common oral flora. Tetanus immunization status was reviewed and found to be up to date. No clinical signs of infection were observed during subsequent evaluation prior to reconstruction.

**FIGURE 2:**
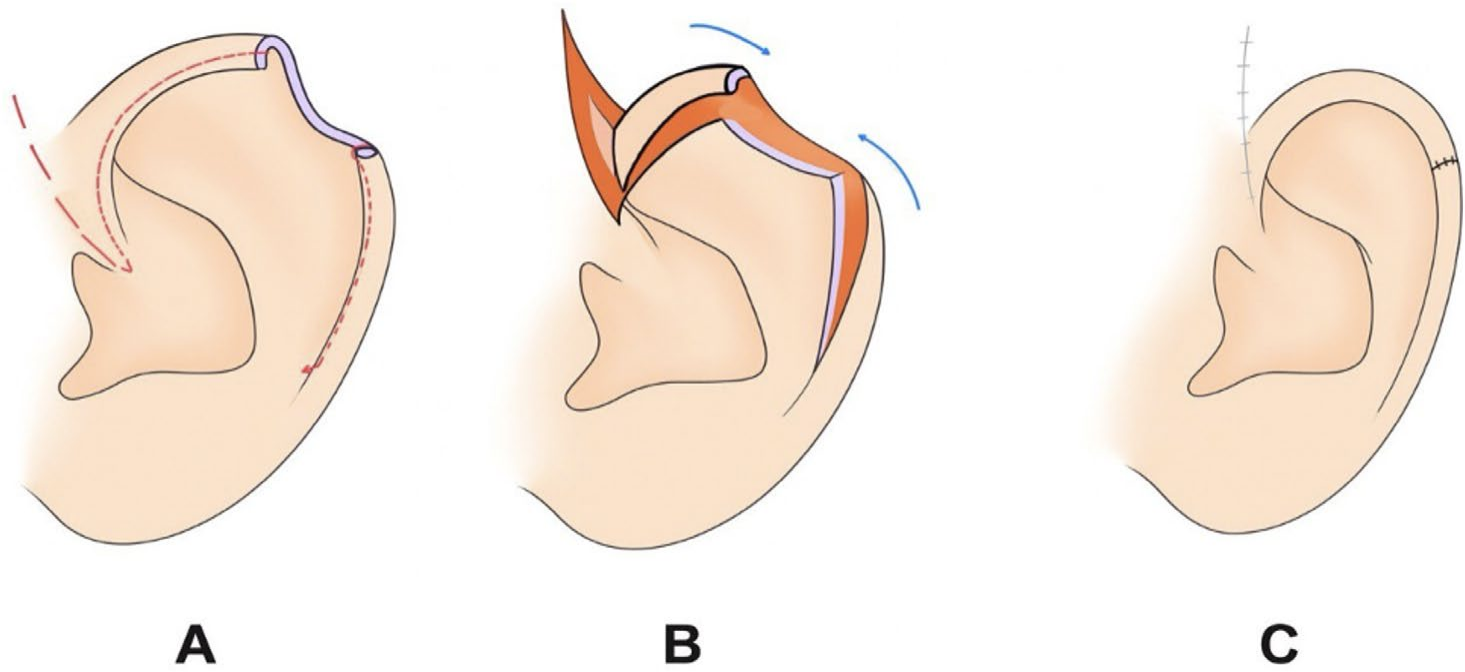
Schematic illustration of the Antia–Buch chondrocutaneous advancement flap used for helical rim reconstruction. (A) Design of the incision along the helical rim. (B) Elevation and advancement of the chondrocutaneous flap. (C) Flap inset and final wound closure.

Several reconstructive options were discussed with the patient, including a single‐stage Antia–Buch chondrocutaneous advancement flap and two‐stage procedures such as bipedicled, tubed, and postauricular flaps. The surgical plan was guided by the patient's preference to preserve ear size and shape, avoid donor‐site skin grafting, and minimize the number of visible surgical stages. On this basis, a modified postauricular flap without skin grafting was selected. The operative design followed the key principles of the Antia–Buch chondrocutaneous advancement flap, including incision planning along the helical rim, flap elevation, advancement, and inset (Figure 2).

**FIGURE 3:**
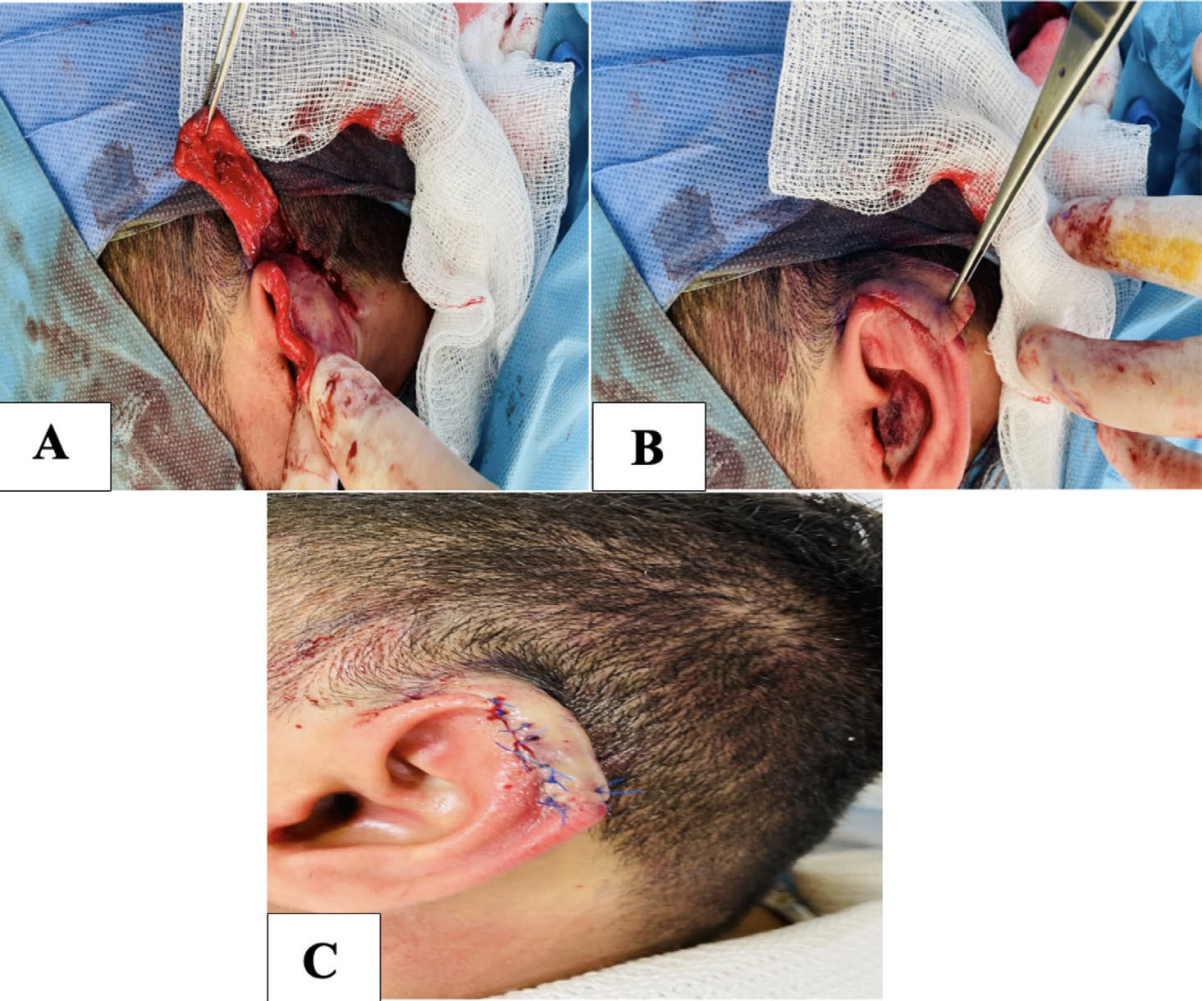
Intraoperative and immediate postoperative views of the modified postauricular flap. (A) Raising and positioning of the postauricular flap onto the helical rim defect. (B) Coverage of the defect following flap advancement. (C) Immediate postoperative appearance showing successful flap inset and restoration of the helical contour.

Intraoperatively, the defect was carefully evaluated and confirmed to involve a middle‐third helical rim defect with preserved surrounding cartilage framework. A modified postauricular flap was designed on the posterior auricular skin with a superiorly based pedicle to preserve vascular supply from the posterior auricular arterial network. The flap dimensions were tailored to match the defect while allowing tension‐free rotation. Dissection was performed in the subcutaneous plane with careful preservation of the vascular pedicle. The flap was then rotated and advanced to cover the helical rim defect. Minor trimming of the cartilage edges was performed to achieve smooth contour alignment. The flap was inset using interrupted absorbable sutures for deep support and fine nylon sutures for skin closure to recreate the natural curvature of the helical rim and prevent notching. The donor site in the postauricular region was closed primarily, preserving the postauricular sulcus (Figure 3A,B). Immediate postoperative appearance demonstrated satisfactory flap adaptation and coverage of the defect with preservation of the surrounding tissue and ear anatomy (Figure 3C).

### 2.3 Outcome and Follow‐Up

**FIGURE 4:**
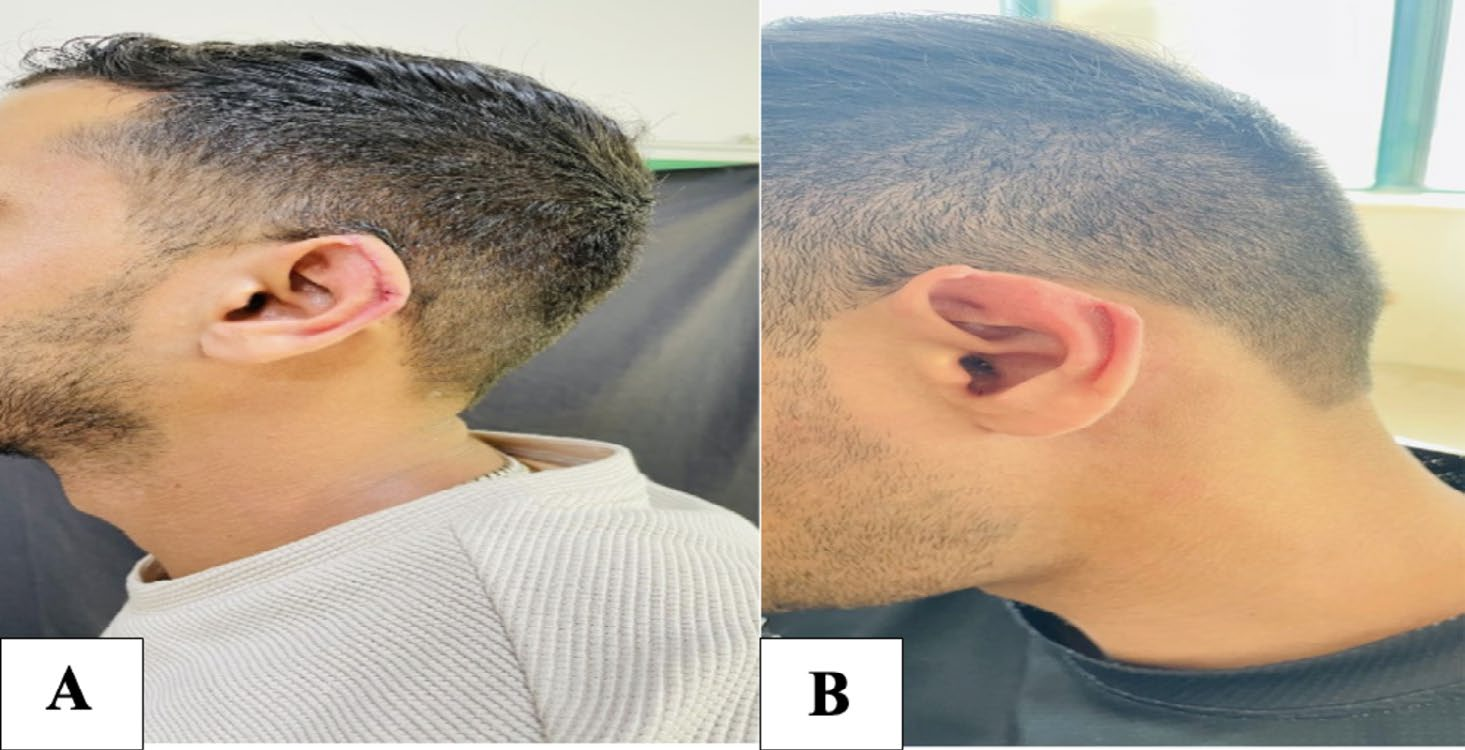
Long‐term postoperative outcome after modified postauricular flap reconstruction. (A) Preoperative view demonstrating the helical rim defect. (B) Postoperative view showing stable auricular contour, healed incision lines, and preserved ear symmetry.

Follow‐up evaluations were performed at 1 week, 1 month, 3 months, and 6 months postoperatively. At the final follow‐up visit, the reconstructed helical rim demonstrated stable contour and preserved auricular symmetry. No complications such as infection, hematoma, partial flap necrosis, hypertrophic scarring, or contour distortion were observed. The patient reported high satisfaction with the aesthetic outcome and symmetry compared with the contralateral ear (Figure 4A,B).

## 3 Discussion

Traumatic injuries of the external ear pose unique reconstructive challenges because of the complex three‐dimensional architecture of the auricle and the limited availability of local tissue for reconstruction. Human bite injuries are particularly problematic due to the high risk of infection, bacterial contamination, and subsequent tissue necrosis, which can compromise the success of primary repair and necessitate secondary reconstruction [1, 2]. In addition, the helical rim plays a key role in maintaining the structural contour and aesthetic balance of the ear; therefore, reconstruction must restore both form and symmetry to achieve satisfactory outcomes [3].

Several surgical techniques have been described for the reconstruction of helical rim defects. Among these, the Antia–Buch chondrocutaneous advancement flap remains one of the most widely accepted approaches for small to moderate marginal defects because it allows advancement of adjacent chondrocutaneous tissue while preserving the natural curvature of the helix [4]. However, this technique may shorten the auricular circumference and can be less suitable for larger defects or for patients who wish to maintain the original ear dimensions [5]. In such situations, regional flaps, including postauricular flaps and temporoparietal fascia flaps, may provide additional tissue coverage while preserving auricular contour and vascular reliability [5, 6, 7].

Postauricular flaps have long been used in auricular reconstruction because of their robust vascular supply, favorable tissue characteristics, and the ability to conceal donor‐site scars within the postauricular sulcus. Various modifications of postauricular flaps have been described to address defects of the helix, antihelix, and conchal regions, offering reliable coverage with good aesthetic outcomes [5, 7, 8]. Compared with staged reconstruction techniques such as tubed or interpolation flaps, single‐stage postauricular flap approaches may reduce treatment duration, minimize patient morbidity, and improve overall satisfaction.

In the present case, a modified postauricular flap was selected based on both the anatomical characteristics of the defect and the patient's preferences. The defect involved the middle third of the helical rim and was associated with necrosis of previously sutured tissue following a human bite injury. The use of a single‐stage flap provided well‐vascularized tissue for reconstruction while preserving the overall size and contour of the auricle. The postoperative outcome demonstrated stable healing, restoration of helical architecture, and high patient satisfaction without evidence of complications such as infection, flap necrosis, or contour distortion.

Management of traumatic ear defects should always take into account the biological behavior of bite wounds, including the risk of infection and delayed tissue viability. Thorough wound evaluation, appropriate antimicrobial management, and careful selection of reconstructive technique are essential for achieving optimal outcomes [1, 2]. In traumatic auricular avulsion injuries, reconstructive planning should also consider tissue viability, defect size, available surgical expertise, and the feasibility of immediate or delayed reconstruction [9]. Furthermore, individualized reconstructive planning that integrates defect size, cartilage integrity, and patient expectations remains a cornerstone of successful auricular reconstruction. For larger helical rim defects or defects with cartilage loss, local flaps, cartilage grafting, or combined reconstructive approaches may be required to restore auricular contour and structural support [10].

Despite the favorable outcome in this case, several limitations should be acknowledged. This report describes a single patient, and therefore, the findings may not be generalizable to all traumatic auricular defects. Variations in defect size, cartilage involvement, wound contamination, and patient factors may influence the choice of reconstructive technique and the outcome. Nevertheless, this case demonstrates that a single‐stage modified postauricular flap can be a reliable and effective option for reconstruction of traumatic helical rim defects in appropriately selected patients.

This case demonstrates that a single‐stage modified postauricular flap can provide reliable reconstruction of traumatic helical rim defects following human bite injuries while preserving auricular contour, ear size, and patient satisfaction. Careful consideration of defect characteristics and patient preferences allowed successful restoration of both function and appearance without the need for staged procedures or donor‐site skin grafting. This patient‐centered approach may be useful for managing similar complex auricular injuries.

## Author Contributions

**Khaled Mohamed AlAani:** conceptualization, data curation, funding acquisition, investigation, methodology, project administration, resources, software, writing – original draft, writing – review and editing. **Ahmed Alanzi:** conceptualization, data curation, formal analysis, funding acquisition, investigation, methodology, project administration, resources, software, supervision, validation, writing – original draft, writing – review and editing. **Madhusudana Naik Mude:** conceptualization, methodology, supervision, validation, visualization, writing – original draft, writing – review and editing. **Dawood Alatefi:** conceptualization, data curation, investigation, methodology, supervision, validation, writing – original draft, writing – review and editing. **Ahmed M. Kazerooni:** resources, software, validation, visualization, writing – original draft. **Ali Mahran Kazerooni:** resources, validation, visualization, writing – original draft. **Noor Mohamed AlAani:** investigation, resources, visualization, writing – original draft.

## Funding

The authors have nothing to report.

## Consent

Written informed consent for publication of this case report and accompanying clinical images was obtained directly from the patient.

## Data Availability

The data used to support the findings of this study are included in the article.
